# Comment on “Depth of Anesthesia as a Risk Factor for Perioperative Morbidity”

**DOI:** 10.1155/2015/301291

**Published:** 2015-11-11

**Authors:** Marco Cascella

**Affiliations:** Department of Anesthesia, Endoscopy and Cardiology, Istituto Nazionale Tumori ‘Fondazione G. Pascale', IRCCS, 80100 Naples, Italy

Petsiti and colleagues recent study on causal relationship between depth of anesthesia and perioperative morbidity [1] is an attractive opportunity to discuss a significant topic: what do we know about Deep Hypnotic Status (DHS) during general anesthesia (GA) and how can we recognize it? In truth, though significant steps have been taken by neuroscience and the knowledge of pharmacodynamics has been fine-tuned, we do not have a comprehensive view of what really happens to the brain during GA. The real problem is driving the anesthesia in a middle earth, furthest from both the burst suppression and sudden emergencies. Thus, if we cannot define the exact anesthesia level of our patient, how can we establish a correlation between DHS and postoperative complications and morbidity? While the authors correctly cited previous studies on the topic, nevertheless we meet Sigl [2] among the authors of one of these references. It is not a big surprise for the scholars of brain monitoring because he was, along with Chamoun, the first to describe the bispectral (BIS) technology in 1994 [3].

It is questionable whether BIS monitor exactly expresses the depth of anesthesia (DOA). However, it may be impossible to measure the real DOA by the simple method accepted by all anesthesiologists. Thus, BIS monitor is regarded as the most reliable and clinically available monitoring for the DOA, although its efficacy is controversial [4].

An anesthesia status not sufficiently deep may cause events of AA; however according to Petsiti et al. a deep hypnotic time (DHT) is a new significant factor associated with postoperative outcome [1]. Petsiti et al. defined DHT as the cumulative period of BIS index values < 45 [1], in line with the previous observations of Lindholm et al. [5]. While single data are not indicative of the anesthesia status, the variable time adds more power to the BIS value. This is the most important issue of the paper by Petsiti et al. because it shows the efficacy of DHT as a risk factor for perioperative mortality, even if the BIS monitoring system is questionable [1].

BIS monitor was the first electroencephalography- (EEG-) based DOA monitor. It is the most widely used system to assess the monitoring of DOA; nevertheless during the last 15–20 years a number of EEG-based technologies have become commercially available. Although several algorithms have been used for patenting brain monitors and many indices are used as references for monitoring the DOA at the moment, there is no device to assess the exact level of anesthesia. Jensen and colleagues have long studied level of consciousness during GA. They published on the detrended fluctuation analysis of EEG, proponing several indexes in order to characterize the patient state (awake, sedated, and anesthetized states) [6]. Nevertheless, as stated by them, these indices are able to detect the loss of consciousness but not to assess the exact level of anesthesia. In the meantime, others have proposed complex algorithms combining EEG analysis and no EEG parameters. Schneider et al. [7] studied a combination of standard monitoring, EEG parameters, and patient and drug information demonstrating that it is possible to separate the different anesthetic levels. However, their pattern is a complex analysis with a late construction which is difficult to apply in order to immediately recognize intraoperative awakening, in particular under neuromuscular blockade. They concluded that “additional studies are required to validate results of the current study.” Recently, the authors of the Michigan Awareness Control Study [8] concluded that they were not able to find an alert threshold for both anesthetic drugs and BIS values and there is the necessity for an individualized anesthetic strategy.

Fortunately, as we are writing the progress of science does not stop. Thus, we are witnessing a rapid evolution of the brain monitoring EEG-based techniques. The studies of Boly et al. [9] on the spectral EEG changes after propofol administration are very important, and Purdon et al. [10] published their fascinating research, explaining that EEG pattern (and its changing) is indicative in real time of the patient transaction from consciousness to the anesthesia status. We hope that these technologies are perfected and soon on a large scale distributed to permanently solve the AA problem and cut down the postoperative complications due to DHT. At the same time the attempts as those of Petsiti are much appreciated.
